# Integration of Spatial and Temporal Patterning in the Invertebrate and Vertebrate Nervous System

**DOI:** 10.3389/fnins.2022.854422

**Published:** 2022-03-22

**Authors:** Yen-Chung Chen, Nikolaos Konstantinides

**Affiliations:** ^1^Department of Biology, New York University, New York, NY, United States; ^2^Université de Paris, Centre National de la Recherche Scientifique, Institut Jacques Monod, Paris, France

**Keywords:** neuronal diversity, spatial patterning, temporal patterning, evolution of developmental mechanisms, fate specification, *Drosophila*, vertebrates

## Abstract

The nervous system is one of the most sophisticated animal tissues, consisting of thousands of interconnected cell types. How the nervous system develops its diversity from a few neural stem cells remains a challenging question. Spatial and temporal patterning mechanisms provide an efficient model through which diversity can be generated. The molecular mechanism of spatiotemporal patterning has been studied extensively in *Drosophila melanogaster*, where distinct sets of transcription factors define the spatial domains and temporal windows that give rise to different cell types. Similarly, in vertebrates, spatial domains defined by transcription factors produce different types of neurons in the brain and neural tube. At the same time, different cortical neuronal types are generated within the same cell lineage with a specific birth order. However, we still do not understand how the orthogonal information of spatial and temporal patterning is integrated into the progenitor and post-mitotic cells to combinatorially give rise to different neurons. In this review, after introducing spatial and temporal patterning in *Drosophila* and mice, we discuss possible mechanisms that neural progenitors may use to integrate spatial and temporal information. We finally review the functional implications of spatial and temporal patterning and conclude envisaging how small alterations of these mechanisms can lead to the evolution of new neuronal cell types.

## Spatiotemporal Patterning And Cell Fate Determination

Traditionally, neuronal types were classified by the distinct functions that they perform (Masland, 2004; Zeng and Sanes, 2017). Neurons can be categorized by their arborization and projection pattern, the neurotransmitter they use, and their electrophysiology. Neurons can also be categorized by the genes they express. The recent advent of high-throughput sequencing has demonstrated that molecular cell types and morphological/functional cell types are largely consistent (Shekhar et al., 2016; Li et al., 2017; Konstantinides et al., 2018b; Sathyamurthy et al., 2018; Delile et al., 2019; Allen et al., 2020; Özel et al., 2020; Yao et al., 2021). How different types of neurons are generated is one of the central questions in developmental neurobiology; the study of spatiotemporal patterning aims to understand the molecular developmental basis of cell type diversity. By deciphering the logic underpinning the patterning process, we can potentially understand how distinct neuronal functions are encoded genetically. Comparing different patterning mechanisms across species could also shed light on how new cell types evolve to generate a more complex nervous system (Holguera and Desplan, 2018; Konstantinides et al., 2018a).

### Spatial Patterning

**FIGURE 1:**
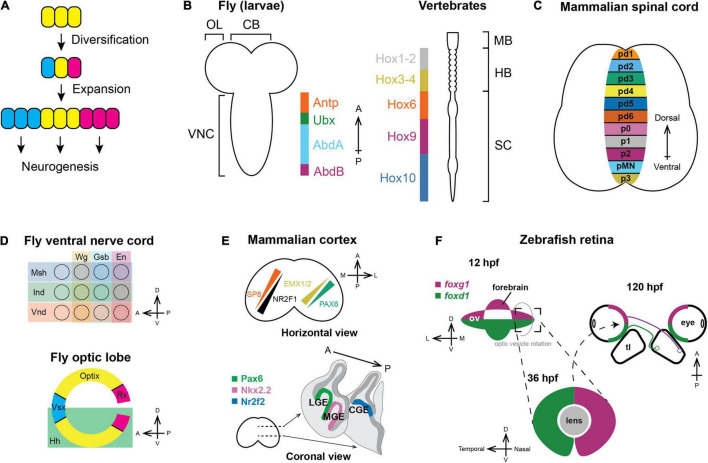
Spatial patterning in neurogenesis. **(A)** General principles of spatial patterning in neurogenesis: Progenitors expand in number and then diversify the set of neuronal types that they can generate during neurogenesis, which is represented by different colors. **(B)** Nervous systems of vertebrates and invertebrates are patterned along the anteroposterior axis by Hox genes. Orthologous Hox genes have the same color (McGinnis and Krumlauf, 1992). (OL: optic lobe; CB: central brain; VNC: ventral nerve cord; MB: midbrain; HB; hindbrain; SC: spinal cord). **(C)** Vertebral neural tubes are patterned along the dorsoventral axis and form multiple progenitor domains that will generate spinal motor neurons and various interneurons (pd1-6: Progenitors for dorsal interneurons dI1-dI6; p0-3: Progenitors for ventral interneuron V0–V3; pMN: Spinal motor neuron progenitors). **(D)** Each segment of the fly ventral nerve cord (top) is partitioned along both the A–P and D–V axis by the expression of spatial factors; similarly, the Outer Proliferation Center neuroepithelium generating the medulla (bottom) is compartmentalized into six distinct domains. **(E)** Upper panel: In mammals, the cortical protomap is defined by the graded expression of transcription factors. SP8 and NR2F1 form opposing gradients where SP8 is highest on the anteromedial side while NR2F1 is highest on the posterolateral side. PAX6 and EMX1/2 form another set of opposing gradients along the anterolateral-posteromedial axis (Reviewed in Greig et al., 2013; Cadwell et al., 2019). Lower panel: Distinct subpallial progenitor domains are marked by specific transcription factors and generate different types of cortical inhibitory interneurons (LGE: Lateral ganglionic eminence; MGE: Medial ganglionic eminence; CGE: Caudal ganglionic eminence). **(F)** In zebrafish, the optic vesicle is dorsoventrally patterned by the expression of *foxg1* and *foxd1*. During developmental eye rotation, the D–V axis becomes the nasotemporal axis, and the nasal hemiretina projects to posterior tectal lobe, while the temporal hemiretina projects to the anterior tectal lobe (hpf: hours post fertilization; ov: optic vesicle; tl: tectal lobe).

Early in animal development, neural progenitors are partitioned into different domains that express distinct transcription factors (Figure 1A). For example, the nervous system of flies and mammals is patterned along the anteroposterior (A–P) axis by Hox genes (Tümpel et al., 2009; Philippidou and Dasen, 2013; Estacio-Gómez and Díaz-Benjumea, 2014; Jung et al., 2014; Figure 1B). Within each segment along the A–P axis, neural progenitors are further patterned on the A-P and dorsoventral (D–V) axis, forming non-intermingling progenitor populations that will generate distinct neuronal types (Jessell, 2000; Briscoe and Ericson, 2001; Karlsson et al., 2010; Benito-Sipos et al., 2013; Birkholz et al., 2013; Figure 1C).

In each segment of the fly ventral nerve cord, neuroepithelial cells are patterned by the expression of *msh*, *ind*, and *vnd* along the D–V axis (Mellerick and Nirenberg, 1995; D’Alessio and Frasch, 1996; Isshiki et al., 1997; Weiss et al., 1998) and *wg*, *gsb*, *en* along the A–P axis (Doe, 1992; Gutjahr et al., 1993; Skeath et al., 1995; Figure 1C). In the fly optic lobe, neuroepithelial cells of the Outer Proliferation Center form a crescent containing six domains defined by Vsx1, Optix, and Rx along the A–P axis and by Hedgehog along the D–V axis (Erclik et al., 2008, 2017; Gold and Brand, 2014; Figure 1D).

In the mammalian forebrain, morphogen gradients pattern radial glia and induce the expression of two orthogonal gradients of spatial transcription factors (Figure 1E, upper panel) that govern the potency of the neural progenitors to generate specific cell types. Different types of cortical interneurons are also made by spatially distinct progenitors. The medial ganglionic eminence expresses Nkx2.1 and is the main source of parvalbumin-and somatostatin-expressing interneurons (Xu, 2004; Flames et al., 2007; Fogarty et al., 2007; Xu et al., 2008), the caudal ganglionic eminence expresses Nr2f2 and generates serotonin-sensing interneurons (Nery et al., 2002; Tripodi et al., 2004; Kanatani et al., 2008; Lee et al., 2010), while the Pax6-expressing lateral ganglionic eminence generates interneurons in the olfactory bulb and the striatum (Wichterle et al., 2001; Kohwi et al., 2007; Figure 1E, lower panel). Another classic example of spatial patterning in vertebrates is the developing retina. In zebrafish, neuroepithelial cells in the optic vesicle are patterned along the D–V axis with foxd1 being expressed in the ventral compartment and foxg1 in the dorsal (Hatini et al., 1994; Yuasa et al., 1996; Picker and Brand, 2005). During anterior eye rotation when the optic cup forms, the D-V spatial domains become the nasal (projecting to the posterior tectum) and temporal (projecting to the anterior tectum) hemiretina, respectively (Figure 1F).

### Temporal Patterning

**FIGURE 2:**
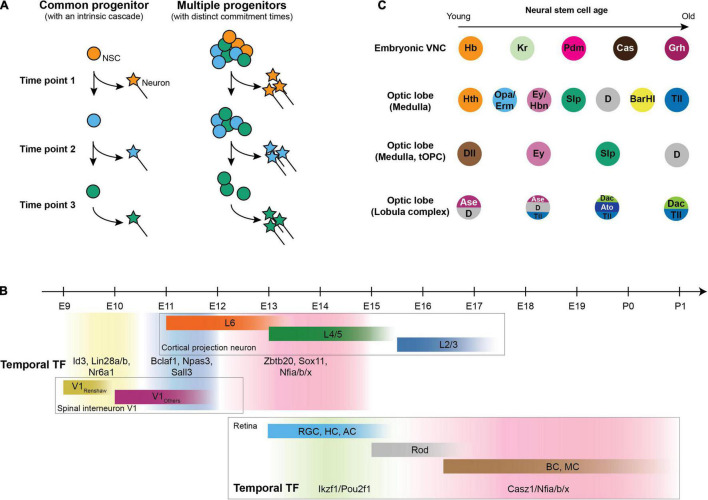
Temporal patterning in neurogenesis. **(A)** Generating distinct neurons at different stages could be either achieved by a common multipotent progenitor that undergoes a cascade (left) or by a pool of progenitors that differ both in potency and in the time to commit to neurogenesis (right). **(B)** Mammalian temporal patterning and neurons generated in each temporal window. Upper panel: Birth date of cortical projection neurons (Caviness, 1982) and V1 spinal interneuron (Stam et al., 2012) with temporal transcription factor expression in neural progenitors at each temporal window (Sagner et al., 2021); lower panel: Birth date of retinal cells and corresponding temporal transcription factors in retinal progenitors [RGC: retinal ganglion cell; HC: horizontal cell; AC: amacrine cell; Rod: rod cell (photoreceptor); BC: bipolar cell; MC: Müller cell] (Elliott et al., 2008; Mattar et al., 2015; Javed et al., 2020). **(C)** Different regions in the fly nervous system undergo different cascades of temporal factors to generate different types of neurons.

The association between time and neural diversity has been known for a long time in mammals. Layers of morphologically distinct neurons in the mammalian cortex are born at specific embryonic days, with each layer projecting to and receiving information from different brain regions (Rakic, 1974; Raedler and Raedler, 1978; Royce, 1983). Two mechanisms could be envisaged to explain how temporal differences result in the generation of distinct neuronal types. First, temporal differences could be intrinsic to each neural progenitor, in which case each progenitor undergoes a cascade of changes to generate different types of neurons at different time points (Figure 2A). Consistent with this model, clonal lineage tracing showed that a single ventricular radial glia cell can generate neurons in all layers (Gao et al., 2014). Alternatively, the neural progenitor pool could be a mixture of different populations that differ in when they start neurogenesis. In this model, progenitors differ both in their potential of generating neurons and the time they commit to neurogenesis (Figure 2A). In support of this model, while the Olig2-expressing progenitors give rise to both early-born spinal motor neurons and late-born oligodendrocytes, there are two subtypes of progenitors: the early ones that generate motor neurons and the ones that are recruited later and only generate oligodendrocytes (Ravanelli and Appel, 2015).

In mammals, recent studies have systematically discovered a set of birthdate markers that are shared between different cell types in the hindbrain and spinal cord of human and mouse (Delile et al., 2019; Osseward et al., 2021; Rayon et al., 2021; Figure 2B), and the expression of these markers are consistent with known subtypes within several cardinal classes of spinal neurons (Roy et al., 2012; Bikoff et al., 2016; Hayashi et al., 2018). Some of these birthdate markers are required for early-vs. late-born neuronal fate in neurons (e.g., in the mammalian cortex, Satb2 is required for later-born callosal neurons from layer 2 to 5, and Fezf2 and Ctip2 are necessary for early-born ones in layer 5) (Chen et al., 2005, 2008; Alcamo et al., 2008; Britanova et al., 2008) and in progenitors (e.g., Nfia and Nfib for the generation of late-born neurons in the retina and ventral spinal interneurons) (Xie et al., 2020; Sagner et al., 2021). Consistent with the idea that neural progenitor temporal factors specify the fate of daughter neurons, cortical neurons inherit the gene modules that are present in radial glia at the stage when the neurons were generated (Telley et al., 2019), and the late-born fate regulators Nfia/b/x directly regulate late-born fate associated genes in the mammalian retina (Clark et al., 2019; Xie et al., 2020; Lyu et al., 2021). Whether it is a general rule that these temporal window-specific gene modules specify the neuronal fates of their daughter cells warrants further investigation.

In the nervous system of flies, cascades of transcription factors expressed in the same neural stem cell underlie temporal patterning, and different regions utilize a distinct set of temporal transcription factors (Brody and Odenwald, 2000; Isshiki et al., 2001; Li et al., 2013; Suzuki et al., 2013; Bertet et al., 2014; Apitz and Salecker, 2015; Erclik et al., 2017; Mora et al., 2018; Pinto-Teixeira et al., 2018; Konstantinides et al., 2021; Zhu et al., 2021; Figure 2C). The temporal cascade in neural stem cells progresses by cross-regulation between temporal factors during neurogenesis, either by a relay of activators (Reviewed in Doe, 2017; Rossi et al., 2017) or by a more robust repressor-decay model (Averbukh et al., 2018). As a result, the expression of each temporal factor in progenitors is responsible for the generation of the neuronal types specific to each temporal window (Isshiki et al., 2001; Mettler et al., 2006; Bayraktar and Doe, 2013; Li et al., 2013; Konstantinides et al., 2021; Tang et al., 2021; Zhu et al., 2021).

## Molecular Logic of Spatiotemporal Integration

The identity of a neural progenitor is defined by the spatial domain from which it originates; the neural progenitor then undergoes a temporal cascade and generates a specific subset of neuronal types within each temporal window of the cascade. How is spatiotemporal identity in progenitors translated to distinct features that define mature neuronal types? In worms, flies, and mammals, the features that define a type of neuron, including neurotransmitter choices and innervation targets, are regulated by post-mitotic regulators that are required and sufficient for the establishment and/or maintenance of the terminal features. For example, CHE-1 binds to a specific motif and defines ASE neuron fate in worms (Uchida et al., 2003; Etchberger et al., 2007), Bsh is required and sufficient for inducing Mi1 neuron fate in the fly optic lobe (Hasegawa et al., 2013), and Ngn2, Isl1, and Lhx3 specify spinal motor neurons in the mammalian spinal cord (Thaler et al., 2002; Lee and Pfaff, 2003; Son et al., 2011; Mazzoni et al., 2013).

For spatiotemporal patterning to specify neuronal types, each spatial and temporal factor must prime the expression of specific post-mitotic fate regulators. Indeed, the *Drosophila* Mi1 neuron is generated during the Hth-expressing temporal window, and the temporal factor Hth is required and sufficient for downstream Bsh expression (Hasegawa et al., 2011; Li et al., 2013). Similarly, Nkx6.1 and Olig2 define the motor neuron progenitor domain in the neural tube and are required and sufficient for the expression of downstream Isl1 and Lhx3 (Sander et al., 2000; Takebayashi et al., 2002).

**FIGURE 3:**
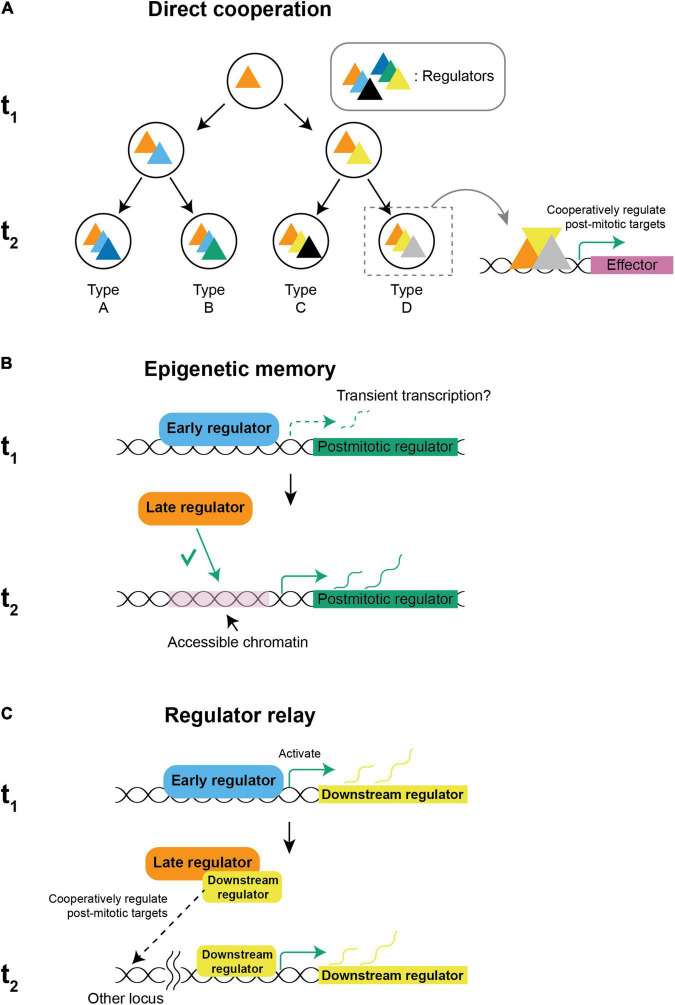
Possible mechanisms of spatiotemporal integration. **(A)** Direct cooperation: New regulators are introduced by an intrinsic cascade or by extrinsic signals and regulate target genes cooperatively with early regulators that are already present to establish distinct gene signatures for different neuronal types. **(B)** Epigenetic memory: Spatial and temporal factors might cooperate by early regulators establishing the chromatin landscape and defining a permissive subset of targets to be regulated by other regulators that are expressed later. The restriction of targets defines a specific set of post-mitotic regulators to be expressed after cell cycle exit. In ASE neurons, transient transcription in early development is sufficient to establish accessibility (Charest et al., 2020). **(C)** Regulator relay: When spatiotemporal factors are not co-expressed, the regulator that is expressed earlier might activate a downstream factor that is persistently expressed. When the later regulator is set to express, it directly cooperates with the downstream factor of the early regulator and activates a specific set of post-mitotic regulators. (t_1_: first time-point, e.g., neural stem cell, t_2_: second time-point, e.g., neuron).

But how do spatial and temporal factors cooperate? Several strategies could be employed:

(1) Spatial and temporal factors could directly cooperate by forming a complex and regulating downstream genes including the post-mitotic regulators (Figure 3A). This strategy is utilized to generate Ap-expressing neurons in the fly embryonic nervous system. Ap^+^ neurons are only present in thoracic segments while the neural stem cells, NB5-6, that generate them are present in every segment. During neurogenesis, Antp, the spatial factor defining the thoracic segments (Figure 1B) and the temporal factors, Cas and Grh (Figure 2C), bind together in a feed-forward loop to activate Col, the post-mitotic regulator for Ap^+^ neurons. The abdominal spatial factors Ubx, Abd-A, and Abd-B together terminate the neurogenesis before the Cas and Grh temporal window to prevent the generation of Ap^+^ neurons in the abdominal segment (Karlsson et al., 2010). *Cis*-regulatory modules targeted by spatiotemporal factors underlie the integration: The *col cis*-regulatory module requires binding by both Antp and Cas to activate, while Antp and Col are required to bind together for Ap and Eya to express and in turn drive the expression of Nplp1, a neuropeptide used by Ap^+^ neurons (Stratmann and Thor, 2017). Extrinsic signals could also introduce regulators to participate in cooperative regulation. For example, in *C. elegans*, AIY neurons are specified by the expression of TTX-3 and CEH-10 (Altun-Gultekin et al., 2001), which depends on iterative integration of the asymmetric Wnt/β-catenin signaling that is only active in the posterior daughter cell (Lin et al., 1998): In a REF-2-expressing progenitor that will generate four types of neurons including an AIY neuron, REF-2 activates *ttx-3* in the anterior daughter cell that is not sensing Wnt (Murgan et al., 2015). When the TTX-3-expressing progenitor divides again, TTX-3 cooperates with β-catenin to activate *ceh*-10 and specify AIY neuron (Bertrand and Hobert, 2009).

(2) However, spatial and temporal factors are not always expressed at the same time, which requires regulators that are expressed at different points to integrate across time. One possibility for an earlier factor to interact with a later factor is by epigenetic memory (Figure 3B). In worms, ASER and ASEL neurons both express the same post-mitotic regulator, CHE-1, but only ASEL expresses LSY-6. The lineages giving rise to ASER/ASEL differ in their early transient expression of TBX-37/38 and become molecularly equivalent after 4 divisions. However, the seemingly equivalent lineages remember their lineage history. Early expression of TBX-37/38 in the ASEL lineage results in transient transcription of *lsy-6*, which is required and sufficient for keeping the locus accessible in the ASEL but not in the ASER lineage, and the difference of accessibility allows CHE-1 to activate *lsy-6* in ASEL but not ASER (Charest et al., 2020). In flies, spatial factors in the ventral nerve cord and optic lobe are expressed in the neuroepithelium and disappear in the neuroblasts, while temporal factors are expressed only in neuroblasts when they commit to neurogenesis. In the ventral nerve cord neuroblasts, chromatin accessibility is regulated differently between spatial domains through the action of a spatial factor, Gsb. Reminiscent to how ASE asymmetry is regulated, the binding pattern of a temporal factor Hb, which is expressed later, is dependent on the pre-existing chromatin landscape and can only bind to the accessible regions (Sen et al., 2019). While chromatin accessibility is determined by multiple factors including histone modifications, DNA methylation (Cusack et al., 2020), pioneer factors (Voss and Hager, 2014), etc., and is used as a proxy of inheritable chromatin modifications, the molecular nature of epigenetic memory remains to be investigated.
(3) Finally, spatiotemporal factors that seem not to be co-expressed could also integrate fate information if the regulator that is expressed earlier turns on a downstream factor that continues to be expressed before eventually cooperating with a later-expressing regulator (Figure 3C).

The models for spatiotemporal integration are not mutually exclusive. On the contrary, spatiotemporal factors are likely to regulate their targets in a hybrid mode. In the fly ventral nerve cord, temporal factors not only regulate their targets directly but could also cooperate with Polycomb complexes to set the epigenetic landscape to restrict the generation of motor neurons to certain temporal windows (Touma et al., 2012). Additionally, genes that are known to be spatiotemporal factors could also play multiple roles in fate specification. For example, in Ap^+^ neurons in the ventral nerve cord, Antp is not only required for the activation of a post-mitotic regulator, Col, but also directly regulates Ap and Eya together with Col, effectively making Antp not only a spatial factor but also a neuronal regulator of post-mitotic features. Finally, while we only used activators as examples in this section, a regulator could serve simultaneously as an activator and a repressor for different sets of targets (Clovis et al., 2016). Therefore, repressors can have a role in all three models: a repressor can suppress the expression of some of the regulators in the complex and change the composition and target genes in the direct cooperation model; a repressive chromatin modifier could silence a locus and prevent later regulators from accessing it in the epigenetic memory model; finally, regulator relay could not only be achieved by an activator cascade but also a sequential decay of repressors (Averbukh et al., 2018).

Our current knowledge of how spatial and temporal patterning are integrated and define cell fates comes from studies of a few genes in a few cell types and is rather limited. The integration of spatial and temporal patterning is more than spatial and temporal factors functioning independently and regulating their own targets. Instead, we know that there are genes that are not activated by either a spatial or a temporal factor alone but only expressed when a specific combination of spatial and temporal factors is present. For example, in the fly optic lobe, Svp is only expressed in neurons generated from the early Hth window from the Vsx and Rx domains but neither Hth nor Vsx or Rx alone is sufficient for the expression of Svp (Erclik et al., 2017). In the ventral nerve cord, Ap neurons are only generated in the Cas window in the thoracic segment defined by Antp (Karlsson et al., 2010). Similarly in vertebrates, Calbindin is only expressed in the early-born V1 interneurons from the p1 progenitor domain but not in early-born neurons from other spatial domains of the spinal cord (Stam et al., 2012). To have a more comprehensive understanding of the molecular logic of spatiotemporal integration, models with known spatial and temporal regulators will be a great entry point, like the fly central nervous system and the mammalian retina and neural tube. To understand the origin of molecular spatiotemporal integration, we need to identify more spatiotemporal factors in various nervous tissues in different species to investigate how these mechanisms have evolved; the advent of high-throughput profiling of gene expression and chromatin landscape specific for each spatiotemporal identity holds the promise to decipher both the rules and exceptions of spatiotemporal integration.

## How Does Spatiotemporal Patterning Contribute to Function?

Spatiotemporal patterning provides an elegant way to generate a wide array of neuronal types that provide the basis for nervous system function. For example, spatial patterning of the neural tube in vertebrates changed concordantly with the emergence of walking in tetrapods and new subtypes of spinal motor neurons were specified to innervate the limbs (Jung et al., 2014, 2018).

Besides providing a gene regulatory framework to enable neurons to acquire their molecular identity, temporal synchronization in neurogenesis helps limit the number of possible targets each neuron encounters and make neural wiring robust and precise during development. Alternatively, temporal patterning allows different types of daughter neurons to be generated next to each other, and this adjacency between daughter neurons could help them to preferentially connect to each other and reduce the number of possible partners by excluding neurons that are farther away. Consistent with this, neurons generated from the same neural progenitor tend to form a circuit, and when each circuit is activated, specific movements, like walking or wing waving, are generated in the fly ventral nerve cord (Harris et al., 2015).

In mammals, the projection pattern of excitatory cortical neurons has long been known to associate with their birthdate (Rakic, 1974; Raedler and Raedler, 1978; Royce, 1983), and clonally related excitatory neurons preferentially connect with each other (Yu et al., 2009, 2012; Li et al., 2012; Ohtsuki et al., 2012). Recently, the organization and projections of spinal neurons were also found to be dependent on their birthdate: Early-born spinal neurons are positioned laterally and enriched in neurons that project a long range, while late-born ones are positioned medially and enriched in neurons that project locally (Osseward et al., 2021). Spatial patterning is also shown to influence multiple neuronal features: Spatial origin is associated with electrophysiological properties of cortical interneurons (Butt et al., 2005) and defines a wide variety of interneurons and motor neurons in the spinal cord (Jessell, 2000; Sagner and Briscoe, 2019). It remains an open question if neurons born at similar stages in different regions of the mammalian nervous system, e.g., the cerebellum and spinal cord, also take advantage of the synchronicity of neurogenesis and preferentially connect and form a circuit among neurons born at similar time windows.

Little is known about the consequences of spatiotemporal patterning dysregulation. In flies, perturbation of temporal patterning by knocking down *eyeless*, an early temporal factor in the central complex, results in impairment of navigation (Sullivan et al., 2019). Similar disruption of navigation is observed when neurons generated during the Eyeless window are silenced (Giraldo et al., 2018; Green et al., 2018; Sullivan et al., 2019). In mice, overproduction of late-born layer 2/3 cortical neurons by prolonging the proliferation of intermediate progenitors results in compulsive behavior and deficits in social behavior reminiscent of autism spectrum disorder (Fang et al., 2013, 2014). Autism spectrum disorder (ASD), a disease involving neocortical function, has been long hypothesized to be associated with miswiring of neural circuitry, and it is tempting to speculate that patterning failure in the cortex results in autistic features if spatiotemporal patterning makes neurogenesis and wiring less error prone. Consistent with this hypothesis, mutations of mammalian cortical layer markers are often associated with ASD (Kwan, 2013). Recently, disorganized cortical lamina and changes in cortical layer thickness were also reported in ASD patients, which indicates that temporal patterning might malfunction during neurogenesis in patients with autism (Wegiel et al., 2010; Stoner et al., 2014; Trutzer et al., 2019).

## Spatiotemporal Patterning and Evolution of New Cell Types

It has long been hypothesized that cell type duplication and diversification underlie nervous system evolution. In this model, the number of neurons expands and then diversifies to serve novel roles to form a more complicated nervous system. Indeed, brain volume and layer thickness differ substantially among vertebrates (Tosches and Laurent, 2019). The gain or loss of spatiotemporal factors is among the plausible mechanisms to diversify an expanded population of neurons. Consistent with this model, recent comparative studies in vertebrates have shown that evolutionary novel subtypes often share the gene expression profile with a conserved cardinal type. For example, birds have only two cerebellar nuclei, medial and interposed, while mammals have three: medial, interposed, and lateral. Neurons from each of the three nuclei in chicken, mice, and humans can be categorized into two molecular classes, while the neurons of the same class from different nuclei still differ from each other (Kebschull et al., 2020). While it remains to be determined if changes in spatiotemporal patterning are responsible for the emergence of new subtypes during evolution, it provides a feasible molecular infrastructure for duplication and diversification to occur.

The recent advent of high-throughput profiling techniques not only made possible the discovery of a shared set of temporal regulators across different vertebrate neuronal tissues (Lu et al., 2020; Sagner et al., 2021) but also opened the opportunity to understand the gene regulatory networks governed by these temporal factors (Telley et al., 2019; Lyu et al., 2021) and other birthdate-specific post-mitotic regulators (Di Bella et al., 2021). Our rapidly expanding knowledge of the molecular logic of spatiotemporal integration and fate specification in various species will set the stage for more comparative studies, such as the investigation of how the targets of conserved spatiotemporal regulators differ between species, how novel spatial domains or temporal windows emerge, and, ultimately, how new cell types are generated during development and evolution.

## Author Contributions

Both authors contributed equally in the conception and writing of the manuscript.

## Conflict of Interest

The authors declare that the research was conducted in the absence of any commercial or financial relationships that could be construed as a potential conflict of interest.

## Publisher’s Note

All claims expressed in this article are solely those of the authors and do not necessarily represent those of their affiliated organizations, or those of the publisher, the editors and the reviewers. Any product that may be evaluated in this article, or claim that may be made by its manufacturer, is not guaranteed or endorsed by the publisher.

## References

[B1] Alcamo E. A. Chirivella L. Dautzenberg M. Dobreva G. Fariñas I. Grosschedl R. (2008). Satb2 regulates callosal projection neuron identity in the developing cerebral cortex. *Neuron* 57 364–377. 10.1016/j.neuron.2007.12.012 18255030

[B2] Allen A. M. Neville M. C. Birtles S. Croset V. Treiber C. D. Waddell S. (2020). A single-cell transcriptomic atlas of the adult *Drosophila* ventral nerve cord. *eLife* 9:e54074. 10.7554/eLife.54074 32314735 PMC7173974

[B3] Altun-Gultekin Z. Andachi Y. Tsalik E. L. Pilgrim D. Kohara Y. Hobert O. (2001). A regulatory cascade of three homeobox genes, ceh-10, ttx-3 and ceh-23, controls cell fate specification of a defined interneuron class in *C. elegans*. *Development* 128 1951–1969. 10.1242/dev.128.11.1951 11493519

[B4] Apitz H. Salecker I. (2015). A region-specific neurogenesis mode requires migratory progenitors in the *Drosophila* visual system. *Nat. Neurosci.* 18 46–55. 10.1038/nn.3896 25501037 PMC4338547

[B5] Averbukh I. Lai S.-L. Doe C. Q. Barkai N. (2018). A repressor-decay timer for robust temporal patterning in embryonic *Drosophila* neuroblast lineages. *eLife* 7:e38631. 10.7554/eLife.38631 30526852 PMC6303102

[B6] Bayraktar O. A. Doe C. Q. (2013). Combinatorial temporal patterning in progenitors expands neural diversity. *Nature* 498 449–455. 10.1038/nature12266 23783519 PMC3941985

[B7] Benito-Sipos J. Baumgardt M. Thor S. (2013). “Chapter 31 - development of the *Drosophila* embryonic ventral nerve cord: from neuroectoderm to unique neurons and glia,” in *Patterning and Cell Type Specification in the Developing CNS and PNS*, eds Rubenstein J. L. R. Rakic P. (Oxford: Academic Press), 627–644. 10.1016/b978-0-12-397265-1.00073-3

[B8] Bertet C. Li X. Erclik T. Cavey M. Wells B. Desplan C. (2014). Temporal patterning of neuroblasts controls notch-mediated cell survival through regulation of hid or reaper. *Cell* 158 1173–1186. 10.1016/j.cell.2014.07.045 25171415 PMC4153738

[B9] Bertrand V. Hobert O. (2009). Linking asymmetric cell division to the terminal differentiation program of postmitotic neurons in *C. elegans*. *Dev. Cell* 16 563–575. 10.1016/j.devcel.2009.02.011 19386265 PMC2691723

[B10] Bikoff J. B. Gabitto M. I. Rivard A. F. Drobac E. Machado T. A. Miri A. (2016). Spinal inhibitory interneuron diversity delineates variant motor microcircuits. *Cell* 165 207–219. 10.1016/j.cell.2016.01.027 26949184 PMC4808435

[B11] Birkholz O. Vef O. Rogulja-Ortmann A. Berger C. Technau G. M. (2013). Abdominal-B and caudal inhibit the formation of specific neuroblasts in the *Drosophila* tail region. *Development* 140 3552–3564. 10.1242/dev.096099 23903193 PMC3915569

[B12] Briscoe J. Ericson J. (2001). Specification of neuronal fates in the ventral neural tube. *Curr. Opin. Neurobiol.* 11 43–49. 10.1016/S0959-4388(00)00172-0 11179871

[B13] Britanova O. de Juan Romero C. Cheung A. Kwan K. Y. Schwark M. Gyorgy A. (2008). Satb2 is a postmitotic determinant for upper-layer neuron specification in the neocortex. *Neuron* 57 378–392. 10.1016/j.neuron.2007.12.028 18255031

[B14] Brody T. Odenwald W. F. (2000). Programmed transformations in neuroblast gene expression during *Drosophila* CNS lineage development. *Dev. Biol.* 226 34–44. 10.1006/dbio.2000.9829 10993672

[B15] Butt S. J. B. Fuccillo M. Nery S. Noctor S. Kriegstein A. Corbin J. G. (2005). The Temporal and Spatial Origins of Cortical Interneurons Predict Their Physiological Subtype. *Neuron* 48 591–604. 10.1016/j.neuron.2005.09.034 16301176

[B16] Cadwell C. R. Bhaduri A. Mostajo-Radji M. A. Keefe M. G. Nowakowski T. J. (2019). Development and arealization of the cerebral cortex. *Neuron* 103 980–1004. 10.1016/j.neuron.2019.07.009 31557462 PMC9245854

[B17] Caviness V. S. (1982). Neocortical histogenesis in normal and reeler mice: a developmental study based upon [3H]thymidine autoradiography. *Dev. Brain Res.* 4 293–302. 10.1016/0165-3806(82)90141-9 7104762

[B18] Charest J. Daniele T. Wang J. Bykov A. Mandlbauer A. Asparuhova M. (2020). Combinatorial action of temporally segregated transcription factors. *Dev. Cell* 55 483.e7–499.e7. 10.1016/j.devcel.2020.09.002 33002421 PMC7704111

[B19] Chen B. Schaevitz L. R. McConnell S. K. (2005). Fezl regulates the differentiation and axon targeting of layer 5 subcortical projection neurons in cerebral cortex. *Proc. Natl. Acad. Sci. U.S.A.* 102 17184–17189. 10.1073/pnas.0508732102 16284245 PMC1282569

[B20] Chen B. Wang S. S. Hattox A. M. Rayburn H. Nelson S. B. McConnell S. K. (2008). The Fezf2-Ctip2 genetic pathway regulates the fate choice of subcortical projection neurons in the developing cerebral cortex. *Proc. Natl. Acad. Sci. U.S.A.* 105 11382–11387. 10.1073/pnas.0804918105 18678899 PMC2495013

[B21] Clark B. S. Stein-O’Brien G. L. Shiau F. Cannon G. H. Davis-Marcisak E. Sherman T. (2019). Single-cell RNA-Seq analysis of retinal development identifies nfi factors as regulating mitotic exit and late-born cell specification. *Neuron* 102 1111.e5–1126.e5. 10.1016/j.neuron.2019.04.010 31128945 PMC6768831

[B22] Clovis Y. M. Seo S. Y. Kwon J. Rhee J. C. Yeo S. Lee J. W. (2016). Chx10 consolidates V2a interneuron identity through two distinct gene repression modes. *Cell Rep.* 16 1642–1652. 10.1016/j.celrep.2016.06.100 27477290 PMC8109239

[B23] Cusack M. King H. W. Spingardi P. Kessler B. M. Klose R. J. Kriaucionis S. (2020). Distinct contributions of DNA methylation and histone acetylation to the genomic occupancy of transcription factors. *Genome Res.* 30 1393–1406. 10.1101/gr.257576.119 32963030 PMC7605266

[B24] D’Alessio M. Frasch M. (1996). msh may play a conserved role in dorsoventral patterning of the neuroectoderm and mesoderm. *Mech. Dev.* 58 217–231. 10.1016/S0925-4773(96)00583-7 8887329

[B25] Delile J. Rayon T. Melchionda M. Edwards A. Briscoe J. Sagner A. (2019). Single cell transcriptomics reveals spatial and temporal dynamics of gene expression in the developing mouse spinal cord. *Development* 146:dev173807. 10.1242/dev.173807 30846445 PMC6602353

[B26] Di Bella D. J. Habibi E. Stickels R. R. Scalia G. Brown J. Yadollahpour P. (2021). Molecular logic of cellular diversification in the mouse cerebral cortex. *Nature* 595 554–559. 10.1038/s41586-021-03670-5 34163074 PMC9006333

[B27] Doe C. Q. (1992). Molecular markers for identified neuroblasts and ganglion mother cells in the *Drosophila* central nervous system. *Development* 116 855–863. 10.1242/dev.116.4.855 1295739

[B28] Doe C. Q. (2017). Temporal patterning in the *Drosophila* CNS. *Annu. Rev. Cell Dev. Biol.* 33 219–240. 10.1146/annurev-cellbio-111315-125210 28992439

[B29] Elliott J. Jolicoeur C. Ramamurthy V. Cayouette M. (2008). Ikaros confers early temporal competence to mouse retinal progenitor cells. *Neuron* 60 26–39. 10.1016/j.neuron.2008.08.008 18940586

[B30] Erclik T. Hartenstein V. Lipshitz H. D. McInnes R. R. (2008). Conserved role of the vsx genes supports a monophyletic origin for bilaterian visual systems. *Curr. Biol.* 18 1278–1287. 10.1016/j.cub.2008.07.076 18723351

[B31] Erclik T. Li X. Courgeon M. Bertet C. Chen Z. Baumert R. (2017). Integration of temporal and spatial patterning generates neural diversity. *Nature* 541 365–370. 10.1038/nature20794 28077877 PMC5489111

[B32] Estacio-Gómez A. Díaz-Benjumea F. J. (2014). Roles of Hox genes in the patterning of the central nervous system of *Drosophila*. *Fly* 8 26–32. 10.4161/fly.27424 24406332 PMC3974890

[B33] Etchberger J. F. Lorch A. Sleumer M. C. Zapf R. Jones S. J. Marra M. A. (2007). The molecular signature and cis-regulatory architecture of a *C. elegans* gustatory neuron. *Genes Dev.* 21 1653–1674. 10.1101/gad.1560107 17606643 PMC1899474

[B34] Fang W.-Q. Chen W.-W. Fu A. K. Y. Ip N. Y. (2013). Axin directs the amplification and differentiation of intermediate progenitors in the developing cerebral cortex. *Neuron* 79 665–679. 10.1016/j.neuron.2013.06.017 23972596

[B35] Fang W.-Q. Chen W.-W. Jiang L. Liu K. Yung W.-H. Fu A. K. Y. (2014). Overproduction of upper-layer neurons in the neocortex leads to autism-like features in mice. *Cell Rep.* 9 1635–1643. 10.1016/j.celrep.2014.11.003 25466248

[B36] Flames N. Pla R. Gelman D. M. Rubenstein J. L. R. Puelles L. Marin O. (2007). Delineation of multiple subpallial progenitor domains by the combinatorial expression of transcriptional codes. *J. Neurosci.* 27 9682–9695. 10.1523/JNEUROSCI.2750-07.2007 17804629 PMC4916652

[B37] Fogarty M. Grist M. Gelman D. Marin O. Pachnis V. Kessaris N. (2007). Spatial genetic patterning of the embryonic neuroepithelium generates GABAergic interneuron diversity in the adult cortex. *J. Neurosci.* 27 10935–10946. 10.1523/JNEUROSCI.1629-07.2007 17928435 PMC6672847

[B38] Gao P. Postiglione M. P. Krieger T. G. Hernandez L. Wang C. Han Z. (2014). Deterministic progenitor behavior and unitary production of neurons in the neocortex. *Cell* 159 775–788. 10.1016/j.cell.2014.10.027 25417155 PMC4225456

[B39] Giraldo Y. M. Leitch K. J. Ros I. G. Warren T. L. Weir P. T. Dickinson M. H. (2018). Sun navigation requires compass neurons in *Drosophila*. *Curr. Biol.* 28 2845.e4–2852.e4. 10.1016/j.cub.2018.07.002 30174187 PMC7301569

[B40] Gold K. S. Brand A. H. (2014). Optix defines a neuroepithelial compartment in the optic lobe of the *Drosophila* brain. *Neural Dev.* 9:18. 10.1186/1749-8104-9-18 25074684 PMC4127074

[B41] Green J. Vijayan V. Mussells Pires P. Adachi A. Maimon G. (2018). Walking *Drosophila* aim to maintain a neural heading estimate at an internal goal angle. *bioRxiv* [Preprint]. 10.1101/315796 PMC7688015 31332373

[B42] Greig L. C. Woodworth M. B. Galazo M. J. Padmanabhan H. Macklis J. D. (2013). Molecular logic of neocortical projection neuron specification, development and diversity. *Nat. Rev. Neurosci.* 14 755–769. 10.1038/nrn3586 24105342 PMC3876965

[B43] Gutjahr T. Patel N. H. Li X. Goodman C. S. Noll M. (1993). Analysis of the gooseberry locus in *Drosophila embryos*: gooseberry determines the cuticular pattern and activates gooseberry neuro. *Development* 118 21–31. 10.1242/dev.118.1.21 8375335

[B44] Harris R. M. Pfeiffer B. D. Rubin G. M. Truman J. W. (2015). Neuron hemilineages provide the functional ground plan for the *Drosophila* ventral nervous system. *eLife* 4:e04493. 10.7554/eLife.04493 26193122 PMC4525104

[B45] Hasegawa E. Kaido M. Takayama R. Sato M. (2013). Brain-specific-homeobox is required for the specification of neuronal types in the *Drosophila* optic lobe. *Dev. Biol.* 377 90–99. 10.1016/j.ydbio.2013.02.012 23454478

[B46] Hasegawa E. Kitada Y. Kaido M. Takayama R. Awasaki T. Tabata T. (2011). Concentric zones, cell migration and neuronal circuits in the *Drosophila* visual center. *Development* 138 983–993. 10.1242/dev.058370 21303851

[B47] Hatini V. Tao W. Lai E. (1994). Expression of winged helix genes, BF-1 and BF-2, define adjacent domains within the developing forebrain and retina. *J. Neurobiol.* 25 1293–1309. 10.1002/neu.480251010 7815060

[B48] Hayashi M. Hinckley C. A. Driscoll S. P. Moore N. J. Levine A. J. Hilde K. L. (2018). Graded arrays of spinal and supraspinal V2a interneuron subtypes underlie forelimb and hindlimb motor control. *Neuron* 97 869.e5–884.e5. 10.1016/j.neuron.2018.01.023 29398364 PMC8601153

[B49] Holguera I. Desplan C. (2018). Neuronal specification in space and time. *Science* 362 176–180. 10.1126/science.aas9435 30309944 PMC6368964

[B50] Isshiki T. Pearson B. Holbrook S. Doe C. Q. (2001). *Drosophila* neuroblasts sequentially express transcription factors which specify the temporal identity of their neuronal progeny. *Cell* 106 511–521. 10.1016/S0092-8674(01)00465-2 11525736

[B51] Isshiki T. Takeichi M. Nose A. (1997). The role of the msh homeobox gene during *Drosophila* neurogenesis: implication for the dorsoventral specification of the neuroectoderm. *Development* 124 3099–3109. 10.1242/dev.124.16.3099 9272951

[B52] Javed A. Mattar P. Lu S. Kruczek K. Kloc M. Gonzalez-Cordero A. (2020). Pou2f1 and Pou2f2 cooperate to control the timing of cone photoreceptor production in the developing mouse retina. *Development* 147:dev188730. 10.1242/dev.188730 32878923

[B53] Jessell T. M. (2000). Neuronal specification in the spinal cord: inductive signals and transcriptional codes. *Nat. Rev. Genet.* 1 20–29. 10.1038/35049541 11262869

[B54] Jung H. Baek M. D’Elia K. P. Boisvert C. Currie P. D. Tay B.-H. (2018). The ancient origins of neural substrates for land walking. *Cell* 172 667.e15–682.e15. 10.1016/j.cell.2018.01.013 29425489 PMC5808577

[B55] Jung H. Mazzoni E. O. Soshnikova N. Hanley O. Venkatesh B. Duboule D. (2014). Evolving hox activity profiles govern diversity in locomotor systems. *Dev. Cell* 29 171–187. 10.1016/j.devcel.2014.03.008 24746670 PMC4024207

[B56] Kanatani S. Yozu M. Tabata H. Nakajima K. (2008). COUP-TFII is preferentially expressed in the caudal ganglionic eminence and is involved in the caudal migratory stream. *J. Neurosci.* 28 13582–13591. 10.1523/JNEUROSCI.2132-08.2008 19074032 PMC6671763

[B57] Karlsson D. Baumgardt M. Thor S. (2010). Segment-specific neuronal subtype specification by the integration of anteroposterior and temporal cues. *PLoS Biol.* 8:e1000368. 10.1371/journal.pbio.1000368 20485487 PMC2867937

[B58] Kebschull J. M. Richman E. B. Ringach N. Friedmann D. Albarran E. Kolluru S. S. (2020). Cerebellar nuclei evolved by repeatedly duplicating a conserved cell-type set. *Science* 370:eabd5059. 10.1126/science.abd5059 33335034 PMC8510508

[B59] Kohwi M. Petryniak M. A. Long J. E. Ekker M. Obata K. Yanagawa Y. (2007). A subpopulation of olfactory bulb GABAergic interneurons is derived from Emx1- and Dlx5/6-expressing progenitors. *J. Neurosci.* 27 6878–6891. 10.1523/JNEUROSCI.0254-07.2007 17596436 PMC4917362

[B60] Konstantinides N. Kapuralin K. Fadil C. Barboza L. Satija R. Desplan C. (2018b). Phenotypic convergence: distinct transcription factors regulate common terminal features. *Cell* 174 622.e13–635.e13. 10.1016/j.cell.2018.05.021 29909983 PMC6082168

[B61] Konstantinides N. Degabriel S. Desplan C. (2018a). Neuro-evo-devo in the single cell sequencing era. *Curr. Opin. Syst. Biol.* 11 32–40. 10.1016/j.coisb.2018.08.001 30886939 PMC6419771

[B62] Konstantinides N. Rossi A. M. Escobar A. Dudragne L. Chen Y.-C. Tran T. (2021). A comprehensive series of temporal transcription factors in the fly visual system. *bioRxiv* [Preprint] 10.1101/2021.06.13.448242 PMC9074256 35388222

[B63] Kwan K. Y. (2013). Transcriptional dysregulation of neocortical circuit assembly in ASD. *Int. Rev. Neurobiol.* 113 167–205. 10.1016/B978-0-12-418700-9.00006-X 24290386 PMC4106301

[B64] Lee S. Hjerling-Leffler J. Zagha E. Fishell G. Rudy B. (2010). The largest group of superficial neocortical GABAergic interneurons expresses ionotropic serotonin receptors. *J. Neurosci.* 30 16796–16808. 10.1523/JNEUROSCI.1869-10.2010 21159951 PMC3025500

[B65] Lee S.-K. Pfaff S. L. (2003). Synchronization of neurogenesis and motor neuron specification by direct coupling of bHLH and homeodomain transcription factors. *Neuron* 38 731–745. 10.1016/S0896-6273(03)00296-4 12797958

[B66] Li H. Horns F. Xie Q. Xie Q. Li T. Luginbuhl D. J. (2017). Classifying *Drosophila* olfactory projection neuron subtypes by single-cell RNA sequencing. *Cell* 171 1206–1207. 10.1016/j.cell.2017.10.019 29149607 PMC6095479

[B67] Li X. Erclik T. Bertet C. Chen Z. Voutev R. Venkatesh S. (2013). Temporal patterning of *Drosophila medulla* neuroblasts controls neural fates. *Nature* 498 456–462. 10.1038/nature12319 23783517 PMC3701960

[B68] Li Y. Lu H. Cheng P. Ge S. Xu H. Shi S.-H. (2012). Clonally related visual cortical neurons show similar stimulus feature selectivity. *Nature* 486 118–121. 10.1038/nature11110 22678292 PMC3375857

[B69] Lin R. Hill R. J. Priess J. R. (1998). POP-1 and anterior–posterior fate decisions in *C. elegans* embryos. *Cell* 92 229–239. 10.1016/S0092-8674(00)80917-4 9458047

[B70] Lu Y. Shiau F. Yi W. Lu S. Wu Q. Pearson J. D. (2020). Single-cell analysis of human retina identifies evolutionarily conserved and species-specific mechanisms controlling development. *Dev. Cell* 53 473.e9–491.e9. 10.1016/j.devcel.2020.04.009 32386599 PMC8015270

[B71] Lyu P. Hoang T. Santiago C. P. Thomas E. D. Timms A. E. Appel H. (2021). Gene regulatory networks controlling temporal patterning, neurogenesis, and cell-fate specification in mammalian retina. *Cell Rep.* 37:109994. 10.1016/j.celrep.2021.109994 34788628 PMC8642835

[B72] Masland R. H. (2004). Neuronal cell types. *Curr. Biol.* 14 R497–R500. 10.1016/j.cub.2004.06.035 15242626

[B73] Mattar P. Ericson J. Blackshaw S. Cayouette M. (2015). A conserved regulatory logic controls temporal identity in mouse neural progenitors. *Neuron* 85 497–504. 10.1016/j.neuron.2014.12.052 25654255 PMC5912935

[B74] Mazzoni E. O. Mahony S. Closser M. Morrison C. A. Nedelec S. Williams D. J. (2013). Synergistic binding of transcription factors to cell-specific enhancers programs motor neuron identity. *Nat. Neurosci.* 16 1219–1227. 10.1038/nn.3467 23872598 PMC3820498

[B75] McGinnis W. Krumlauf R. (1992). Homeobox genes and axial patterning. *Cell* 68 283–302. 10.1016/0092-8674(92)90471-N 1346368

[B76] Mellerick D. M. Nirenberg M. (1995). Dorsal-ventral patterning genes restrict NK-2 homeobox gene expression to the ventral half of the central nervous system of *Drosophila embryos*. *Dev. Biol.* 171 306–316. 10.1006/dbio.1995.1283 7556915

[B77] Mettler U. Vogler G. Urban J. (2006). Timing of identity: spatiotemporal regulation of hunchback in neuroblast lineages of *Drosophila* by seven-up and prospero. *Development* 133 429–437. 10.1242/dev.02229 16396905

[B78] Mora N. Oliva C. Fiers M. Ejsmont R. Soldano A. Zhang T. T. (2018). A temporal transcriptional switch governs stem cell division, neuronal numbers, and maintenance of differentiation. *Dev. Cell* 45 53.e5–66.e5. 10.1016/j.devcel.2018.02.023 29576424

[B79] Murgan S. Kari W. Rothbächer U. Iché-Torres M. Mélénec P. Hobert O. (2015). Atypical transcriptional activation by TCF via a zic transcription factor in *C. elegans* neuronal precursors. *Dev. Cell* 33 737–745. 10.1016/j.devcel.2015.04.018 26073017 PMC4480195

[B80] Nery S. Fishell G. Corbin J. G. (2002). The caudal ganglionic eminence is a source of distinct cortical and subcortical cell populations. *Nat. Neurosci.* 5 1279–1287. 10.1038/nn971 12411960

[B81] Ohtsuki G. Nishiyama M. Yoshida T. Murakami T. Histed M. Lois C. (2012). Similarity of visual selectivity among clonally related neurons in visual cortex. *Neuron* 75 65–72. 10.1016/j.neuron.2012.05.023 22794261

[B82] Osseward P. J. Amin N. D. Moore J. D. Temple B. A. Barriga B. K. Bachmann L. C. (2021). Conserved genetic signatures parcellate cardinal spinal neuron classes into local and projection subsets. *Science* 372 385–393. 10.1126/science.abe0690 33888637 PMC8612134

[B83] Özel M. N. Simon F. Jafari S. Holguera I. Chen Y.-C. Benhra N. (2020). Neuronal diversity and convergence in a visual system developmental atlas. *Nature* 589 88–95. 10.1038/s41586-020-2879-3 33149298 PMC7790857

[B84] Philippidou P. Dasen J. S. (2013). Hox genes: choreographers in neural development, architects of circuit organization. *Neuron* 80 12–34. 10.1016/j.neuron.2013.09.020 24094100 PMC3835187

[B85] Picker A. Brand M. (2005). Fgf signals from a novel signaling center determine axial patterning of the prospective neural retina. *Development* 132 4951–4962. 10.1242/dev.02071 16236770

[B86] Pinto-Teixeira F. Koo C. Rossi A. M. Neriec N. Bertet C. Li X. (2018). Development of concurrent retinotopic maps in the fly motion detection circuit. *Cell* 173 485.e11–498.e11. 10.1016/j.cell.2018.02.053 29576455 PMC5889347

[B87] Raedler E. Raedler A. (1978). Autoradiographic study of early neurogenesis in rat neocortex. *Anat. Embryol.* 154 267–284. 10.1007/BF00345657 707818

[B88] Rakic P. (1974). Neurons in rhesus monkey visual cortex: systematic relation between time of origin and eventual disposition. *Science* 183 425–427. 10.1126/science.183.4123.425 4203022

[B89] Ravanelli A. M. Appel B. (2015). Motor neurons and oligodendrocytes arise from distinct cell lineages by progenitor recruitment. *Genes Dev.* 29 2504–2515. 10.1101/gad.271312.115 26584621 PMC4691953

[B90] Rayon T. Maizels R. J. Barrington C. Briscoe J. (2021). Single cell transcriptome profiling of the human developing spinal cord reveals a conserved genetic programme with human specific features. *bioRxiv* [Preprint]. 10.1101/2021.04.12.439474 PMC8353162 34351410

[B91] Rossi A. M. Fernandes V. M. Desplan C. (2017). Timing temporal transitions during brain development. *Curr. Opin. Neurobiol.* 42 84–92. 10.1016/j.conb.2016.11.010 27984764 PMC5316342

[B92] Roy A. Francius C. Rousso D. L. Seuntjens E. Debruyn J. Luxenhofer G. (2012). Onecut transcription factors act upstream of Isl1 to regulate spinal motoneuron diversification. *Development* 139 3109–3119. 10.1242/dev.078501 22833130 PMC4074251

[B93] Royce G. J. (1983). Cortical neurons with collateral projections to both the caudate nucleus and the centromedian-parafascicular thalamic complex: a fluorescent retrograde double labeling study in the cat. *Exp. Brain Res.* 50 157–165. 10.1007/BF00239179 6196226

[B94] Sagner A. Briscoe J. (2019). Establishing neuronal diversity in the spinal cord: a time and a place. *Development* 146:dev182154. 10.1242/dev.182154 31767567

[B95] Sagner A. Zhang I. Watson T. Lazaro J. Melchionda M. Briscoe J. (2021). A shared transcriptional code orchestrates temporal patterning of the central nervous system. *PLoS Biol.* 19:e3001450. 10.1371/journal.pbio.3001450 34767545 PMC8612522

[B96] Sander M. Paydar S. Ericson J. Briscoe J. Berber E. German M. (2000). Ventral neural patterning by Nkx homeobox genes: Nkx6.1 controls somatic motor neuron and ventral interneuron fates. *Genes Dev.* 14 2134–2139. 10.1101/gad.820400 10970877 PMC316892

[B97] Sathyamurthy A. Johnson K. R. Matson K. J. E. Dobrott C. I. Li L. Ryba A. R. (2018). Massively parallel single nucleus transcriptional profiling defines spinal cord neurons and their activity during behavior. *Cell Rep.* 22 2216–2225. 10.1016/j.celrep.2018.02.003 29466745 PMC5849084

[B98] Sen S. Q. Chanchani S. Southall T. D. Doe C. Q. (2019). Neuroblast-specific open chromatin allows the temporal transcription factor, Hunchback, to bind neuroblast-specific loci. *eLife* 8:e44036. 10.7554/eLife.44036 30694180 PMC6377230

[B99] Shekhar K. Lapan S. W. Whitney I. E. Tran N. M. Macosko E. Z. Kowalczyk M. (2016). Comprehensive classification of retinal bipolar neurons by single-cell transcriptomics. *Cell* 166 1308.e30–1323.e30. 10.1016/j.cell.2016.07.054 27565351 PMC5003425

[B100] Skeath J. B. Zhang Y. Holmgren R. Carroll S. B. Doe C. Q. (1995). Specification of neuroblast identity in the *Drosophila* embryonic central nervous system by gooseberry-distal. *Nature* 376 427–430. 10.1038/376427a0 7630418

[B101] Son E. Y. Ichida J. K. Wainger B. J. Toma J. S. Rafuse V. F. Woolf C. J. (2011). Conversion of mouse and human fibroblasts into functional spinal motor neurons. *Cell Stem Cell* 9 205–218. 10.1016/j.stem.2011.07.014 21852222 PMC3188987

[B102] Stam F. J. Hendricks T. J. Zhang J. Geiman E. J. Francius C. Labosky P. A. (2012). Renshaw cell interneuron specialization is controlled by a temporally restricted transcription factor program. *Development* 139 179–190. 10.1242/dev.071134 22115757 PMC3231776

[B103] Stoner R. Chow M. L. Boyle M. P. Sunkin S. M. Mouton P. R. Roy S. (2014). Patches of disorganization in the neocortex of children with autism. *N. Engl. J. Med.* 370 1209–1219. 10.1056/NEJMoa1307491 24670167 PMC4499461

[B104] Stratmann J. Thor S. (2017). Neuronal cell fate specification by the molecular convergence of different spatio-temporal cues on a common initiator terminal selector gene. *PLoS Genet.* 13:e1006729. 10.1371/journal.pgen.1006729 28414802 PMC5411104

[B105] Sullivan L. F. Warren T. L. Doe C. Q. (2019). Temporal identity establishes columnar neuron morphology, connectivity, and function in a *Drosophila* navigation circuit. *eLife* 8:e43482. 10.7554/eLife.43482 30706848 PMC6386519

[B106] Suzuki T. Kaido M. Takayama R. Sato M. (2013). A temporal mechanism that produces neuronal diversity in the *Drosophila* visual center. *Dev. Biol.* 380 12–24. 10.1016/j.ydbio.2013.05.002 23665475

[B107] Takebayashi H. Nabeshima Y. Yoshida S. Chisaka O. Ikenaka K. Nabeshima Y. (2002). The basic helix-loop-helix factor Olig2 is essential for the development of motoneuron and oligodendrocyte lineages. *Curr. Biol.* 12 1157–1163. 10.1016/S0960-9822(02)00926-0 12121626

[B108] Tang J. L. Y. Hakes A. E. Krautz R. Suzuki T. Contreras E. G. Fox P. M. (2021). NanoDam identifies novel temporal transcription factors conserved between the *Drosophila* central brain and visual system. *Dev. Biol.* [Epub ahead of print]. 10.1101/2021.06.07.447332 PMC9616798 35483359

[B109] Telley L. Agirman G. Prados J. Amberg N. Fièvre S. Oberst P. (2019). Temporal patterning of apical progenitors and their daughter neurons in the developing neocortex. *Science* 364:eaav2522. 10.1126/science.aav2522 31073041

[B110] Thaler J. P. Lee S.-K. Jurata L. W. Gill G. N. Pfaff S. L. (2002). LIM factor Lhx3 contributes to the specification of motor neuron and interneuron identity through cell-type-specific protein-protein interactions. *Cell* 110 237–249. 10.1016/S0092-8674(02)00823-1 12150931

[B111] Tosches M. A. Laurent G. (2019). Evolution of neuronal identity in the cerebral cortex. *Curr. Opin. Neurobiol.* 56 199–208. 10.1016/j.conb.2019.04.009 31103814

[B112] Touma J. J. Weckerle F. F. Cleary M. D. (2012). *Drosophila* Polycomb complexes restrict neuroblast competence to generate motoneurons. *Development* 139 657–666. 10.1242/dev.071589 22219354

[B113] Tripodi M. Filosa A. Armentano M. Studer M. (2004). The COUP-TF nuclear receptors regulate cell migration in the mammalian basal forebrain. *Development* 131 6119–6129. 10.1242/dev.01530 15548577

[B114] Trutzer I. M. García-Cabezas M. Á Zikopoulos B. (2019). Postnatal development and maturation of layer 1 in the lateral prefrontal cortex and its disruption in autism. *Acta Neuropathol. Commun.* 7:40. 10.1186/s40478-019-0684-8 30867066 PMC6417186

[B115] Tümpel S. Wiedemann L. M. Krumlauf R. (2009). Chapter 8 hox genes and segmentation of the vertebrate hindbrain. *Curr. Top. Dev. Biol.* 88 103–137. 10.1016/S0070-2153(09)88004-6 19651303

[B116] Uchida O. Nakano H. Koga M. Ohshima Y. (2003). The C. elegans che-1 gene encodes a zinc finger transcription factor required for specification of the ASE chemosensory neurons. *Development* 130 1215–1224. 10.1242/dev.00341 12588839

[B117] Voss T. C. Hager G. L. (2014). Dynamic regulation of transcriptional states by chromatin and transcription factors. *Nat. Rev. Genet.* 15 69–81. 10.1038/nrg3623 24342920 PMC6322398

[B118] Wegiel J. Kuchna I. Nowicki K. Imaki H. Wegiel J. Marchi E. (2010). The neuropathology of autism: defects of neurogenesis and neuronal migration, and dysplastic changes. *Acta Neuropathol.* 119 755–770. 10.1007/s00401-010-0655-4 20198484 PMC2869041

[B119] Weiss J. B. Von Ohlen T. Mellerick D. M. Dressler G. Doe C. Q. Scott M. P. (1998). Dorsoventral patterning in the *Drosophila* central nervous system: the intermediate neuroblasts defective homeobox gene specifies intermediate column identity. *Genes Dev.* 12 3591–3602. 10.1101/gad.12.22.3591 9832510 PMC317240

[B120] Wichterle H. Turnbull D. H. Nery S. Fishell G. Alvarez-Buylla A. (2001). In utero fate mapping reveals distinct migratory pathways and fates of neurons born in the mammalian basal forebrain. *Development* 128 3759–3771. 10.1242/dev.128.19.3759 11585802

[B121] Xie H. Zhang W. Zhang M. Akhtar T. Li Y. Yi W. (2020). Chromatin accessibility analysis reveals regulatory dynamics of developing human retina and hiPSC-derived retinal organoids. *Sci. Adv.* 6:eaay5247. 10.1126/sciadv.aay5247 32083182 PMC7007246

[B122] Xu Q. (2004). Origins of cortical interneuron subtypes. *J. Neurosci.* 24 2612–2622. 10.1523/JNEUROSCI.5667-03.2004 15028753 PMC6729522

[B123] Xu Q. Tam M. Anderson S. A. (2008). Fate mapping Nkx2.1-lineage cells in the mouse telencephalon. *J. Comp. Neurol.* 506 16–29. 10.1002/cne.21529 17990269

[B124] Yao Z. van Velthoven C. T. J. Nguyen T. N. Goldy J. Sedeno-Cortes A. E. Baftizadeh F. (2021). A taxonomy of transcriptomic cell types across the isocortex and hippocampal formation. *Cell* 184 3222.e26–3241.e26. 10.1016/j.cell.2021.04.021 34004146 PMC8195859

[B125] Yu Y.-C. Bultje R. S. Wang X. Shi S.-H. (2009). Specific synapses develop preferentially among sister excitatory neurons in the neocortex. *Nature* 458 501–504. 10.1038/nature07722 19204731 PMC2727717

[B126] Yu Y.-C. He S. Chen S. Fu Y. Brown K. N. Yao X.-H. (2012). Preferential electrical coupling regulates neocortical lineage-dependent microcircuit assembly. *Nature* 486 113–117. 10.1038/nature10958 22678291 PMC3599787

[B127] Yuasa J. Hirano S. Yamagata M. Noda M. (1996). Visual projection map specified by topographic expression of transcription factors in the retina. *Nature* 382 632–635. 10.1038/382632a0 8757134

[B128] Zeng H. Sanes J. R. (2017). Neuronal cell-type classification: challenges, opportunities and the path forward. *Nat. Rev. Neurosci.* 18 530–546. 10.1038/nrn.2017.85 28775344

[B129] Zhu H. Zhao S. D. Ray A. Zhang Y. Li X. (2021). A comprehensive temporal patterning gene network in *Drosophila* medulla neuroblasts revealed by single-cell RNA sequencing. *bioRxiv* [Preprint]. 10.1101/2021.06.12.448145 PMC8913700 35273186

